# Virulence potential of *Rickettsia amblyommatis* for spotted fever pathogenesis in mice

**DOI:** 10.1093/femspd/ftab024

**Published:** 2021-04-28

**Authors:** Wan-Yi Yen, Kayla Stern, Smruti Mishra, Luke Helminiak, Santiago Sanchez-Vicente, Hwan Keun Kim

**Affiliations:** Division of Laboratory Animal Resources, Laboratory of Comparative Medicine, Stony Brook University, Stony Brook, NY 11794, USA; John F. Kennedy High School, Bellmore, NY 11710, USA; Center for Infectious Diseases, Department of Microbiology and Immunology, Stony Brook University, Stony Brook, NY 11794, USA; Center for Infectious Diseases, Department of Microbiology and Immunology, Stony Brook University, Stony Brook, NY 11794, USA; Center for Infectious Diseases, Department of Microbiology and Immunology, Stony Brook University, Stony Brook, NY 11794, USA; Center for Infectious Diseases, Department of Microbiology and Immunology, Stony Brook University, Stony Brook, NY 11794, USA

**Keywords:** *Rickettsia amblyommatis*, *Amblyomma americanum*, spotted fever, pathogenesis, endothelial cell, animal infection model

## Abstract

*Rickettsia amblyommatis* belongs to the spotted fever group of *Rickettsia* and infects *Amblyomma americanum* (Lone Star ticks) for transmission to offspring and mammals. Historically, the geographic range of *A. americanum* was restricted to the southeastern USA. However, recent tick surveys identified the progressive northward invasion of *A. americanum*, contributing to the increased number of patients with febrile illnesses of unknown etiology after a tick bite in the northeastern USA. While serological evidence strongly suggests that patients are infected with *R. amblyommatis*, the virulence potential of *R. amblyommatis* is not well established. Here, we performed a bioinformatic analysis of three genome sequences of *R. amblyommatis* and identified the presence of multiple putative virulence genes whose products are implicated for spotted fever pathogenesis. Similar to other pathogenic spotted fever rickettsiae, *R. amblyommatis* replicated intracellularly within the cytoplasm of tissue culture cells. Interestingly, *R. amblyommatis* displayed defective attachment to microvascular endothelial cells. The attachment defect and slow growth rate of *R. amblyommatis* required relatively high intravenous infectious doses to produce dose-dependent morbidity and mortality in C3H mice. In summary, our results corroborate clinical evidence that *R. amblyommatis* can cause mild disease manifestation in some patients.

## Teaser

*Rickettsia amblyommatis*, an obligate intracellular pathogen transmitted by Lone Star ticks (*Amblyomma americanum*), infects endothelial cells and causes spotted fever pathogenesis in mice.

## INTRODUCTION

Ticks transmit numerous life-threatening infectious diseases that have no preventive vaccines or immune therapeutics and pose ongoing public health threats (Eisen *et al*. 2017). Several environmental and sociological factors have contributed to the expansion and invasion of multiple tick species, resulting in an increased number of tick-borne infections in the USA (Childs and Paddock 2003; Sonenshine 2018). Recent tick surveillance studies have demonstrated that *Amblyomma americanum* (Lone Star tick) has rapidly expanded northward and become the dominant tick species, displacing local tick species such as *Ixodes scapularis* (Deer tick) and *Dermacentor variabilis* (American Dog tick), in the Northeast and Midwest, where an increased number of patients with tick-borne febrile diseases of unknown etiology has been observed (Dahlgren *et al*. 2016; Rosenberg *et al*. 2018; Molaei *et al*. 2019; Sanchez-Vicente *et al*. 2019).

Among the several human pathogens transmitted by *A. americanum*, *Rickettsia amblyommatis* (formerly known as *Candidatus* Rickettsia amblyommii), a Gram-negative pathogen that belongs to the spotted fever group (SFG) of *Rickettsia*, has been identified in a large population of surveyed ticks (Killmaster *et al*. 2014; Karpathy *et al*. 2016; De Jesus *et al*. 2019; Sanchez-Vicente *et al*. 2019). By contrast, the current prevalence of *Rickettsia rickettsii*, the causative agent of Rocky Mountain spotted fever (RMSF), in *D. variabilis* is estimated to be less than 1% (Dahlgren *et al*. 2016; Hecht *et al*. 2019). The high prevalence of *R. amblyommatis*, combined with the aggressive biting behavior of *A. americanum*, enhances the probability of human infections with *R. amblyommatis* (Childs and Paddock 2003). Within ticks, *R. amblyommatis* exhibits obligatory intracellular lifecycle in multiple organ tissues such as ovaries, midgut and salivary glands (Zanetti *et al*. 2008). Infection in these tick organs facilitates the transmission of *R. amblyommatis* to offspring and mammalian hosts (Macaluso *et al*. 2001; Levin, Schumacher and Snellgrove 2018; Suwanbongkot *et al*. 2019). Unlike strictly endosymbiotic *Rickettsia* species (e.g. *Rickettsia buchneri* in *I. scapularis*), several recently characterized pathogenic *Rickettsia* species, such as *Rickettsia helvetica*, *Rickettsia slovaca* and *Rickettsia parkeri*, have also been identified in the salivary glands and midgut of ticks, tested for their virulence using *in vitro* and *in vivo* infection models, then isolated from humans after tick bites, corroborating that the presence of *Rickettsia* in the salivary glands in ticks facilitates transmission to mammalian hosts (Raoult *et al*. 2002; Paddock *et al*. 2004; Nilsson, Elfving and Pahlson 2010; Zemtsova *et al*. 2016; Engstrom *et al*. 2019; Pahlson *et al*. 2020).

The hallmark of rickettsial diseases is the invasion of *Rickettsia* into vascular endothelial cells, followed by intracellular replication, vascular pathologies and bacteremia for transmission of *Rickettsia* into uninfected vectors (Hackstadt 1996; Sahni *et al*. 2019). Progressive endothelial cell injury leads to the generation of characteristic erythematous rash, vasculitis, cutaneous necrosis and life-threatening symptoms, including sepsis, making *Rickettsia* one of the most deadly pathogens (Walker and Ismail 2008). Although *R. amblyommatis* has not been isolated from patients, clinical and serological evidence suggests that this microorganism may be the etiological agent of RMSF-like illness in humans. In 2006, a partially engorged female *A. americanum* was removed from a patient who developed a macular rash at the tick bite site (Billeter *et al*. 2007). A PCR test confirmed the presence of *R. amblyommatis* and the absence of *Borrelia*, *Ehrlichia*, *Anaplasma*, *Babesia*, *Bartonella* and other pathogenic *Rickettsia* species (Billeter *et al*. 2007). Analysis of paired sera from patients diagnosed with probable RMSF revealed that some patients developed antibodies to *R. amblyommatis*, but not to *R. rickettsii* and *R. parkeri* (the causative agent of *R. parkeri* rickettsioses), corroborating that *R. amblyommatis* may cause RMSF-like illnesses in humans (Apperson *et al*. 2008; Vaughn *et al*. 2014; Delisle *et al*. 2016). Those patients with specific reactivity to *R. amblyommatis* presented typical clinical manifestations of a mild RMSF with fever, headache and myalgia (Delisle *et al*. 2016). Recent reports found that domestic and wild small mammals (dogs and cats) elicit *R. amblyommatis*-specific antibodies and may serve as natural hosts for *R. amblyommatis* transmission (Barrett, Little and Shaw 2014; Costa *et al*. 2017; Springer *et al*. 2018; Lopes *et al*. 2019).

Investigations into the virulence potential of *R. amblyommatis* generated mixed results. Previous work suggested that *R. amblyommatis* is non-pathogenic as two strains, WB-8–2 and North Texas, failed to cause clinical diseases in guinea pigs (Burgdorfer, Cooney and Thomas 1974; Blanton *et al*. 2014). By contrast, another *R. amblyommatis* strain, 9-CC-3–1, caused vascular inflammation in guinea pigs (Rivas *et al*. 2015). A recent report also documented that intraperitoneal injection of *R. amblyommatis* strain Lake Alexander caused mild disease manifestations in guinea pigs (Snellgrove *et al*. 2021). This is not an unusual finding among *Rickettsia*, as strains of *R. rickettsii* displayed drastically different virulence in animal infection models (Clark *et al*. 2015; Galletti *et al*. 2021). Interestingly, infections with *R. amblyommatis* raised broad-spectrum immune responses and provided cross-immune protection in guinea pigs against virulent *R. rickettsii* infections (Blanton *et al*. 2014; Rivas *et al*. 2015). To better understand the biology of *R. amblyommatis*, we utilized bioinformatic tools, *in vitro* tissue culture assay and *in vivo* animal infection models. Our bioinformatic analysis shows that putative virulence genes whose products are characterized for spotted fever pathogenesis in animal infection models are present in *R. amblyommatis*. Using *in vitro* tissue culture assays, we demonstrate that *R. amblyommatis* strain GAT-30V replicates within the cytoplasm of host cells with defective attachment and invasion into microvascular endothelial cells. While assessing its virulence, we determined relatively high infectious doses that produced significant morbidity and mortality in C3H mice. Together, our results support clinical evidence that *R. amblyommatis* may cause mild rickettsioses in some patients.

## MATERIALS AND METHODS

### Cell lines and bacterial strains

Vero cells (African green monkey kidney cells, ATCC, Manassas, VA) were cultured in Dulbecco's modified Eagle's medium (DMEM, Gibco, Waltham, MA) supplemented with 10% heat-inactivated fetal bovine serum (HI-FBS, HyClone, Marlborough, MA) at 37°C in a 5% CO_2_ atmosphere. HMEC-1 cells (human dermal microvascular endothelial cells, ATCC) were cultured in MCDB 131 medium (Gibco) supplemented with 10 ng∙ml^−1^ epidermal growth factor (Gibco), 1 μg∙ml^−1^ hydrocortisone (Sigma, St. Louis, MO), 10 mM glutamine (Corning, Corning, NY) and 10% HI-FBS at 37°C in a 5% CO_2_ atmosphere. Stocks of *Rickettsia conorii* strain Malish 7 (ATCC VR-613) and *R. amblyommatis* strain GAT-30V (CDC, Dr. Chris Paddock) were generated by growing rickettsiae in Vero cells (5% HI-FBS, DMEM) at 34°C in a 5% CO_2_ atmosphere. Rickettsiae were purified from Vero cells by differential centrifugation through 33% MD-76R solution (816 mM meglumine diatrizoate, 157 mM sodium diatrizoate hydrate, 1 mM NaH_2_PO_4_, pH 7.0; 21 000 × g, 4°C, 20 min) and stored at –80°C in SPG buffer (218 mM sucrose, 3.8 mM KH_2_PO_4_, 7.2 mM K_2_HPO_4_, 4.9 mM L-glutamate, pH 7.2).

### Bioinformatic analysis

Genomic data for *Rickettsia* were collected from the National Center for Biotechnology Information GenBank database (*R. amblyommatis* strain GAT-30V, accession no. CP003334.1; *R. amblyommatis* strain Ac37, accession no. CP012420.1; *R. amblyommatis* strain An13, accession no. CP015012.1; *R. conorii* strain Malish 7, accession no. AE006914.1; *R. rickettsii* strain Sheila Smith, accession no. CP000848.1). Sequences were analyzed with Mauve Multiple Genome Alignment software (ver. snapshot_2015–02–25), Protein BLAST (https://blast.ncbi.nlm.nih.gov/Blast.cgi) and Clustal Omega (https://www.ebi.ac.uk/Tools/msa/clustalo/) to identify conserved and divergent regions in the bacterial genomes, homologs in the designated species and amino acid % identity.

### Growth and plaque assay

Growth curves were generated by infecting *R. conorii* and *R. amblyommatis* into monolayers of Vero or HMEC-1 cells in six-well plates at a multiplicity of infection (MOI) of 0.01. Infected tissue culture cells were incubated at 34°C in a 5% CO_2_ atmosphere. At 1-h post-infection and 2-day intervals, host cells in each well were dislodged with 3-mm glass beads and lysed by vortexing with 3-mm glass beads. The infectious titers were determined by infecting fresh monolayers of Vero cells with 10-fold serial dilutions of lysates containing *Rickettsia* in DMEM supplemented with 5% HI-FBS. Upon infection, Vero cells were incubated at 34°C with 5% CO_2_ for 60 min to allow attachment and overlaid with DMEM containing 5% HI-FBS and 0.5% agarose. The *Rickettsia* attachment and invasion levels were determined by dividing the number of *Rickettsia* infectious titers from the cell lysates by the number of *Rickettsia* infectious titers in the media.

### Microscopy analyses

Cytopathology in Vero and HMEC-1 cell cultures was analyzed by microscopy. On days 2, 4, 6 and 8 post-infection, differential interference contrast (DIC) images of Vero and HMEC-1 cells infected with *R. conorii* or *R. amblyommatis* at a MOI of 0.01 in multiple fields of six-well plates were captured with a 12-bit charge-coupled device camera (Eclipse TE300 fluorescent microscope, Laboratory of Comparative Medicine, Stony Brook University) and contrast-adjusted using ImageJ (NIH). Areas of cytopathic Vero cells were assessed by NIH ImageJ software. For confocal laser scanning microscopic studies, HMEC-1 cells were cultured on a eight-well chamber slide (Lab-Tek, Nunc, Rochester, NY), infected with *R. amblyommatis* at a MOI of 0.01, fixed with 10% formalin for 10 min and post-fixed with 100% methanol for 5 min on Days 4, 6 and 8 of infection. Next, the fixed cells were permeabilized with 0.1% Triton X-100, incubated with mouse anti-*β*-actin antibody (1:500, Sigma) and rabbit anti-*R. conorii* LPS (1:500, crossreacts with SFG *Rickettsia*) overnight (Kim *et al*. 2019). The secondary antibodies (Alexa Fluor 488 donkey anti-mouse [1:1000, Invitrogen, Waltham, MA] and Alexa Fluor 594 donkey anti-rabbit IgG [1:1000, Invitrogen]) were incubated at room temperature for 1 h. The nuclei of HMEC cells were labeled by Hoechst 33 342 (Prolong Glass Antifade Mountant with NucBlue, Invitrogen). The images were taken by Leica SP8 confocal microscope (Advanced Energy Research & Technology Center, Stony Brook University) and analyzed with ImageJ (NIH).

### Mouse model of spotted fever

C3H mice (male and female, 6-week-old, N = 5 per group, Charles River Laboratories, Wilmington, MA) were anesthetized via intraperitoneal injection with 100 mg·ml^−1^ of ketamine and 20 mg·ml^−1^ of xylazine per kilogram of body weight. Animals were infected via intravenous retro-orbital injection with Renografin-purified 5 ‒ 50 × 10^5^ PFU *R. amblyommatis* strain GAT-30V in 0.1 ml SPG buffer or mock-infected with retro-orbital injections of 0.1 ml SPG buffer. Infected mice were monitored twice daily for signs of disease and daily for weight loss. Two days following infection, the heart was removed during necropsy and heart homogenate was analyzed for rickettsial load by plaque assay.

### Whole-cell ELISA

Nunc MaxiSorp 96-well plates were coated with 5 × 10^5^ PFU formalin-inactivated *R. amblyommatis* strain GAT-30V in 0.1 M carbonate buffer (pH 9.5 at 4°C) overnight. The following day, plates were washed three times with 0.05% (v/v) Tween 20 in PBS and blocked with 5% non-fat milk (w/v) in PBS for 1 h at room temperature. For the determination of *R. amblyommatis*-specific IgG titers, ELISA plates were washed and incubated with dilutions of hyperimmune sera for 1 h at room temperature. Following the wash, HRP-conjugated goat anti-mouse IgG (1:10 000, Rockland, Pottstown, PA) antibody was added and incubation was repeated as above. The wells were developed using an OptEIA kit (BD Lifesciences, Franklin Lakes, NJ) and absorbance at 450 nm was measured to calculate half-maximal titers using Prism software (GraphPad, San Diego, CA).

### Biosafety and biosecurity

Animal research was performed in accordance with institutional guidelines following experimental protocol review, approval and supervision by the Institutional Biosafety Committee and the Institutional Animal Care and Use Committee at Stony Brook University. Animals were managed by the Division of Laboratory Animal Resources (Stony Brook University), which is accredited by the American Association for Accreditation of Laboratory Animal Care and the Department of Health and Human Services. Animals were maintained in accordance with the applicable portions of the Animal Welfare Act and the DHHS ‘Guide for the Care and Use of Laboratory Animals’. Veterinary care was under the direction of full-time resident veterinarians boarded by the American College of Laboratory Animal Medicine. Experiments with infectious *Rickettsia* were performed in biosafety level 3 containment.

### Statistical analyses

All statistical analyses were performed using Prism software (GraphPad). Two-tailed Student's *t*-test was performed to calculate the statistical significance of *Rickettsia* attachment and invasion into HMEC-1 cells. Two-way ANOVA with Sidak's multiple comparison test was performed to analyze the statistical significance of body weight change and *Rickettsia* growth data.

### Materials and data availability

The data and unique materials that support the findings of this study are available from the corresponding author upon request.

## RESULTS

### Genetic analysis of *R. amblyommatis*

**Figure 1.**
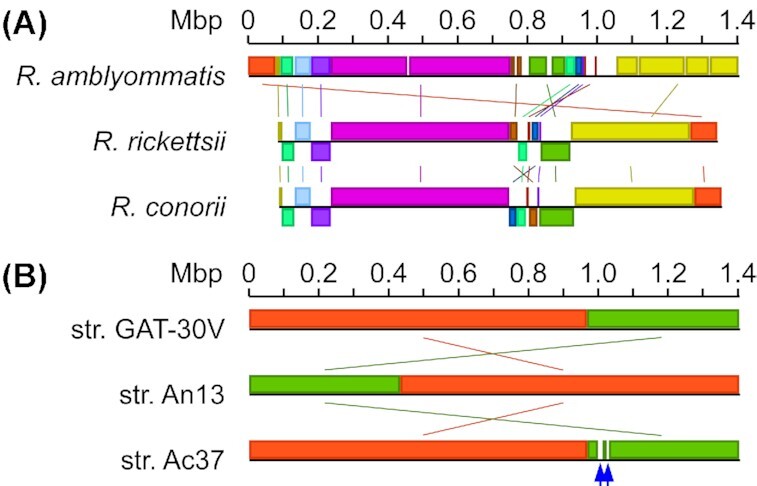
Schematic diagrams of Mauve gene alignments for **(A)***R. amblyommatis* strain GAT-30V (GenBank accession no. CP003334.1), *R. rickettsii* strain Sheila Smith (GenBank accession no. CP000848.1) and *R. conorii* strain Malish 7 (GenBank accession no. AE006914.1) and **(B)***R. amblyommatis* strain GAT-30V (GenBank accession no. CP003334.1), *R. amblyommatis* strain Ac37 (GenBank accession no. CP012420.1) and *R. amblyommatis* strain An13 (GenBank accession no. CP015012.1). Blue arrows point to genetic regions only present in *R. amblyommatis* strain Ac37.

Recent analyses of rickettsial genomes and advances in genetic studies of *Rickettsia* provided insights into the biology of *Rickettsia* with the identification of conserved and unique genes involved in the rickettsial lifecycle (Andersson *et al*. 1998; McLeod *et al*. 2004). The evolution of *Rickettsia* pathogens seems to follow the strategy of genome reduction, where genes are inactivated or lost via deletions, thereby increasing microbial dependence on host metabolism and nutrients while simultaneously increasing the virulence potential (Diop, Raoult and Fournier 2018, 2019). The genome sequence analyses revealed that SFG genomes (∼1.2–1.6 Mbp) encode ∼1200–1500 proteins, with unique gene rearrangements and single nucleotide polymorphisms in each genome (Ogata *et al*. 2005; Matsutani *et al*. 2013; Clark *et al*. 2015; Londono *et al*. 2019). SFG rickettsiae present with different clinical characteristics and severity, suggesting that multiple unique rickettsial and host factors engage in clinical pathogenesis. To determine the genomic similarities between *R. amblyommatis* and other pathogenic SFG rickettsiae, the genetic sequence of *R. amblyommatis* strain GAT-30V was compared with those of *R. rickettsii* strain Sheila Smith and *R. conorii* strain Malish 7. The genome of *R. amblyommatis* GAT-30V consists of a singular chromosome predicted to encode 1229 proteins with 371 pseudogenes and three circular pRM plasmids containing 31 974, 18 263 and 22 851 bp, respectively (Table 1). While *R. rickettsii* and *R. conorii* do not have circular plasmids, their chromosomal DNA sequences are predicted to produce 1234 and 1269 proteins with 150 and 223 pseudogenes (Table 1). Cross-comparison analysis of three spotted fever *Rickettsia* genome sequences identified high degrees of sequence identity (*R. amblyommatis* vs. *R. rickettsii*, 97.03% identity with 88% query coverage; *R. amblyommatis* vs. *R. conorii*, 96.09% identity with 89% query coverage), multiple rearranged genetic fragments and regions lost in *R. conorii* and *R. rickettsii* (Fig. 1A). Further, numerous putative virulence genes that have been characterized for spotted fever pathogenesis are present with 86–95% amino acid identity in *R. amblyommatis* (Table 2) (Gillespie *et al*. 2015). Thus, our bioinformatic analyses suggest that *R. amblyommatis* has the potential to cause disease in mammalian hosts.

**Table 1.** Genomic statistics for *R. amblyommatis*, *R.rickettsii* and *R. conorii*.

*Rickettsia* *^a^*	Total no. of bases	No. of genes	No. of genes encoding proteins	No. of pseudo genes
*R. amblyommatis* str. GAT-30V	1 407 796	1640	1229	371
*R. rickettsii* str. Sheila Smith	1 257 710	1425	1234	150
*R. conorii* str. Malish 7	1 268 755	1532	1269	223

a GenBank accession numbers for *R. amblyommatis* strain GAT-30V, *R. rickettsii* strain Sheila Smith and *R. conorii* strain Malish 7 are CP003334.1, CP000848.1 and AE006914.1, respectively.

**Table 2.** Putative virulence genes conserved in *R. amblyommatis*.

Putative virulence factors^a^	Gene numbers in str. GAT-30V^b^	Gene numbers in str. An13^c^	Gene numbers in str. Ac37^d^	% identity to *R. rickettsii*^e^	Predicted virulence functions^f^
Adr1	MCE_08 045	A3305_02 145	AL573_07 330	94.63%	Complement resistance
Adr2	MCE_08 050	A3305_02 150	AL573_07 335	95.09%	Complement resistance
Pat1	MCE_06 135	A3305_00 385	AL573_05 575	88.57%	Phagolysosome escape
RickA	MCE_06 070	A3305_00 325	AL573_05 520	91.02%	Actin-based motility
RARP2	MCE_06 205	A3305_00 455	AL573_05 645	90.13%	Effector protein of T4SS
Sca0	MCE_07 995	A3305_02 110	AL573_07 295	86.65%	Host cell adhesion
Sca1	MCE_00 840	A3305_02 870	AL573_00 730	87.67%	Host cell adhesion
Sca2	MCE_01 310	A3305_03 305	AL573_01 175	86.69%	Actin-based motility
Sca4	MCE_04 250	A3305_06 025	AL573_03 910	93.89%	Cell-to-cell spread
Sca5	MCE_07 010	A3305_01 180	AL573_06 365	90.27%	Host cell adhesion

a Annotated virulence factors shown to be involved in spotted fever pathogenesis in animal infection models.

b Gene numbers in *R. amblyommatis* strain GAT-30V (GenBank: CP003334.1).

c Gene numbers in *R. amblyommatis* strain An13 (GenBank: CP015012.1).

d Gene numbers in *R. amblyommatis* strain Ac37 (GenBank: CP012420.1).

e Percent identity to amino acids in *R. rickettsii* strain Sheila Smith (GenBank: CP000848.1).

f Predicted virulence functions of each gene products.

### Reductive genome evolution of *R. amblyommatis*

Three genome sequences of *R. amblyommatis* strains are available in the GenBank database. The GAT-30V strain was isolated from *A. americanum* in Georgia, a part of historical regions known for *A. americanum* geographic distribution. The other two *R. amblyommatis* strains, Ac37 and An13, have been isolated from *Amblyomma cajennense* in Brazil and *Amblyomma neumanni* in Argentina. Genetic adaptation to arthropod vectors is predicted to significantly impact vector competence and disease transmission and provide a selective pressure on the genetics of arthropod-borne pathogens (Gooding 1996). Genetic variability among the *R. rickettsii* strains has been characterized for the basis of differences in the virulence and disease severity in animals and humans (Ellison *et al*. 2008). Thus, we cross-compared three genetic sequences of *R. amblyommatis* strains derived from different tick species in three distinct geographical locations to determine the level of genetic stability. While three genomic sequences of *R. amblyommatis* strains displayed high similarity (>99%), we noted three genetic regions that are present in the strain Ac37 but absent in strains GAT-30V and An13 (Fig. 1B). Genes in these regions in *R. amblyommatis* Ac37 appear to be undergoing deletions and frameshifts, representing a hot spot for genome degradation in *R. amblyommatis* (Table 3). Two of the genes in the first region (1 000 184–1 012 987) encode enzymes involved in stringent responses and another encodes IS100 family transposase. Also, there were three non-functional copies of genes predicted to be the remnants of genes encoding transposases and a heat-shock protein. Interestingly, all genes in the second region (1 027 069–1 030 599) are disrupted genes, with a pseudogene homologous to AL573_05 270 that appears in the first region. The last region (1 046 364–1 048 082) only represents one gene, AL573_05 435. Genome reduction has been described for other SFG *Rickettsia* species, such as *R. conorii* and *R. rickettsii* (Ogata *et al*. 2001; Ellison *et al*. 2008). Here, we characterized an ongoing process of genome reduction in *R. amblyommatis*.

**Table 3.** Genes present in *R. amblyommatis* strain AC37.

Gene numbers in *R. amblyommatis* strain Ac37^a^	Coding region^b^	Predicted protein^c^
*First region (1 000 184–1 012 987)*		
AL573_05 270	999 477–1 000 205	Guanosine polyphosphate pyrophosphohydrolase
Pseudo gene	1 000 869–1 000 958	Transposase
AL573_05 280	1 001 169–1 002 161	IS110 family transposase
AL573_05 285	1 002 491–1 005 442	Hypothetical protein
AL573_05 290	1 005 559–1 006 263	GNAT family N-acetyltransferase
AL573_05 295	1 006 359–1 006 973	Bifunctional (p)ppGpp synthetase/guanosine-3',5'-bis(diphosphate) 3'-pyrophosphohydrolase
Pseudo gene	1 007 184–1 007 327	Heat-shock protein
Pseudo gene	1 007 561–1 008 505	Transposase
AL573_05 310	1 008 587–1 008 913	Hypothetical protein
AL573_05 315	1 008 960–1 012 943	Hypothetical protein
AL573_05 320	1 012 943–1 013 182	Hypothetical protein
*Second region (1 027 069–1 030 599)*		
Pseudo gene	1 025 546–1 027 072	Conjugal transfer protein TraG
Pseudo gene	1 027 108–1 027 997	Hypothetical protein
Pseudo gene	1 028 082–1 028 883	DNA methyltransferase
Pseudo gene	1 029 291–1 030 208	Histidine kinase
Pseudo gene	1 030 362–1 031 021	Guanosine polyphosphate pyrophosphohydrolase
*Third region (1 046 364–1 048 082)*		
AL573_05 435	1 045 757–1 049 095	tetratricopeptide repeat protein

a Gene numbers in *R. amblyommatis* strain Ac37 (GenBank accession no. CP012420.1).

b Coding regions annotated in GenBank.

c Prediction of protein-coding genes in GenBank.

### *Rickettsia amblyommatis* is defective for attachment to endothelial cells and replicates at a slower rate

**Figure 2.**
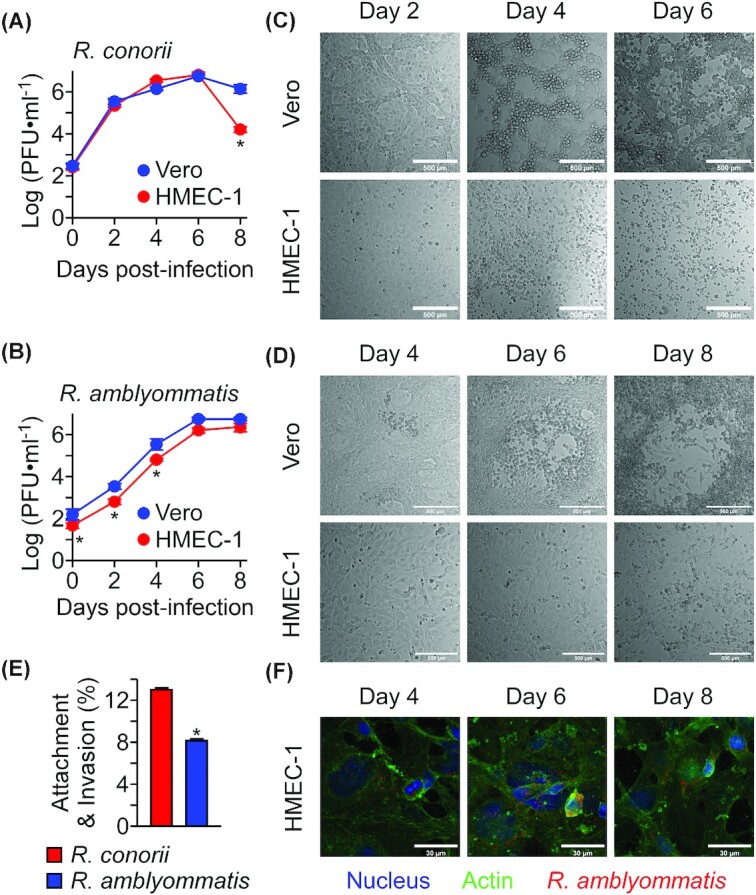
*Rickettsia amblyommatis* is defective for attachment to host endothelial cells and replicates at a slower rate. **(A)***Rickettsia conorii* strain Malish 7 and **(B)***R. amblyommatis* strain GAT-30V replications in Vero and HMEC-1 cells were quantified with the plaque assay (mean ± SEM, N = 3). Representative differential interference contrast (DIC) microscopic images of Vero and HMEC-1 cells infected with **(C)***R. conorii* or **(D)***R. amblyommatis* on 2, 4, 6 and 8 days post-inoculation (scale bar, 500 µm). **(E)***Rickettsia conorii* strain Malish 7 and *R. amblyommatis* strain GAT-30V attachment and invasion into HMEC-1 cells were quantified with the plaque assay on Vero cells (mean ± SEM, N = 3). **(F)** Representative confocal microscopic images of intracellular *R. amblyommatis* in HMEC-1 cells (scale bar, 30 µm). *, *P* < 0.05. Data are representative of two independent experiments.

While *Rickettsia* targets and invades vascular endothelial cells in humans, multiple *in vitro* tissue culture systems have supported rickettsial replication and enabled investigators to identify rickettsial and host factors involved in the rickettsial obligate intracellular lifecycle (Haglund *et al*. 2010; Lehman *et al*. 2018; Kim *et al*. 2019). To characterize *R. amblyommatis* growth phenotypes, we performed pairwise comparisons of bacterial replication at timed intervals in Vero (African green monkey kidney epithelial cells) and HMEC-1 (human dermal microvascular endothelial cells). When inoculated at a multiplicity of infection of 0.01, *R. conorii* Malish 7 expanded rapidly for the first 2 days of infection (Fig. 2A). By day 6, *R. conorii* formed large plaques and caused extensive necrosis and destruction of both cell types (Fig. 2C). On day 8 post-infection, the number of infectious *R. conorii* dropped significantly as no host cells were available to support its obligate intracellular lifecycle on HMEC-1 cells. Importantly, *R. conorii* replicated at a comparable rate in both cell types (*P* <>0.05i>). Next, we performed the same growth analysis with *R. amblyommatis* GAT-30V (Fig. 2B). While both cell types supported *R. amblyommatis* intracellular lifecycle, *R. amblyommatis* replicated at a slower rate (*R. amblyommatis* vs. *R. conorii* on Vero cells at days 2, 4 and 8, *P* <<0.05i>; *R. amblyommatis* vs. *R. conorii* on HMEC-1 cells at days 0, 2, 4, 6 and 8, *P* <<0.01i>, Fig. 2D). Furthermore, *R. amblyommatis* GAT-30V displayed a significant defect in host cell adhesion and invasion on HMEC-1 cells (*R.conorii* vs. *R. amblyommatis*, *P* <0.0001, Fig. 2E). On day 4 of inoculation, *R. amblyommatis* on Vero cells started to form small plaques, which expanded to 249 ± 13 μm (mean ± SEM, N = 20, Day 6) and 964 ± 30 μm (mean ± SEM, N = 20, Day 8, Fig. 2D). In addition to the attachment defect, *R. amblyommatis* produced minimal cytopathology on HMEC-1 cell cultures for 8 days of infection (Fig. 2D and E). These data suggest that *R. amblyommatis* replicates at a slower rate compared with other pathogenic SFG rickettsiae in tissue culture cells, requires specific gene products to survive in endothelial cells and causes minimal cytopathology in endothelial cells.

### Virulence potential of *R. amblyommatis* in C3H mice

**Figure 3.**
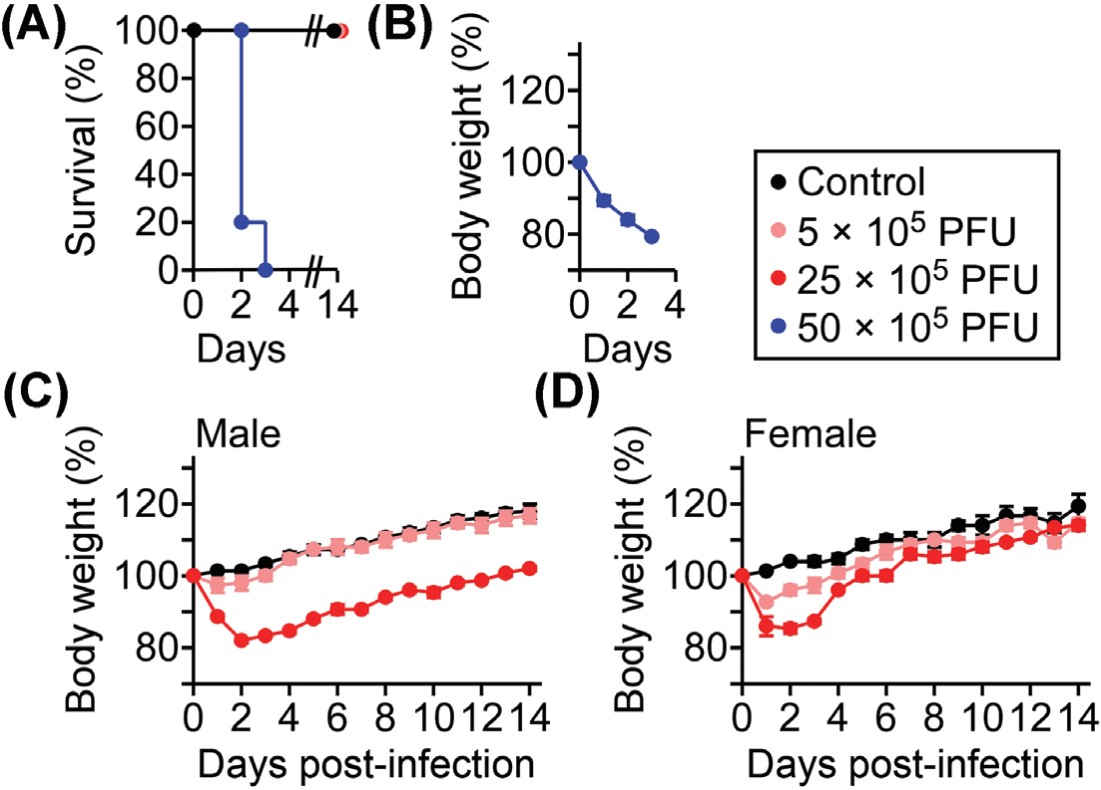
*Rickettsia amblyommatis* causes spotted fever pathogenesis in C3H mice. **(A)** Kaplan-Meier analysis for survival and **(B)** body weight analysis of C3H mice (N = 5) infected with 5–50 × 10^5^ PFU *R. amblyommatis* strain GAT-30V. (**C** and **D**) Body weight analysis of C3H mice (N = 5) infected with 5–25 × 10^5^ PFU of *R. amblyommatis*. Data are representative of two independent experiments.

To investigate whether *R. amblyommatis* exhibits acute diseases in the mouse model for spotted fever, cohorts of C3H mice were intravenously infected with 5–50 × 10^5^ PFU *R. amblyommatis* GAT-30V. Animals infected with 5 × 10^6^ PFU *R. amblyommatis* GAT-30V exhibited severe body weight loss (>20%) and lethal outcome within 3 days of inoculation (Fig. 3A and B). When infected with a sub-lethal dose (5 × 10^5^ PFU), animals exhibited modest body weight losses within 3 days, followed by a fast recovery (Day 3, male, *P* <<0.05i>; Days 1–3, female, *P* <<0.05i>; Fig. 3C and D). On the other hand, animals infected with 25 × 10^5^ PFU of *R. amblyommatis* GAT-30V displayed acute disease with significant body weight loss during the first 2 days of infection followed by a slow recovery for the next 5–10 days (Days 1–14, male, *P* <<0.01i>; Days 1–6, female, *P* <<0.01i>; Fig. 3C and D). Following 2 days of infection, we recovered 90 ± 43 PFU∙ml^−1^ (mean ± SEM, N = 5, male) *R. amblyommatis* from heart homogenates of animals infected with 25 × 10^5^ PFU of *R. amblyommatis*. Further, animals developed *R. amblyommatis*-specific half-maximal IgG titers of 5986 ± 787 (mean ± SEM, N = 5, male) on Day 14 of infection. Taken together, these data suggest that *R. amblyommatis* is responsible for the observed clinical symptoms in mice. Of note, these infectious doses are ~5–2500-fold higher than the experimental lethal (1 × 10^6^ PFU) and sub-lethal (1 × 10^3^ PFU) doses of *R. conorii* Malish 7 determined in the mouse infection model for spotted fever (Kim *et al*. 2019).

## DISCUSSION

Our results demonstrate that *R. amblyommatis* displays defective host cell attachment and invasion phenotype, replicates within host endothelial cells at a slower rate and causes mild spotted fever diseases in mice. Our data corroborate clinical and serological evidence that *R. amblyommatis* may cause self-limiting mild febrile illnesses in humans and other mammals. Thus far, *R. amblyommatis* has not been isolated from patients, and the quantitative and qualitative natures of *R. amblyommatis* transmission during tick blood meal remain unknown (Esteves *et al*. 2019). Cytotoxic T lymphocyte activities against infected host cells and complement-mediated bacterial killing modulate host-protective immune responses against rickettsial infections (Walker, Olano and Feng 2001; Riley *et al*. 2018). Recent studies highlight the critical roles of inflammasome and interferons in fighting against infections caused by *Rickettsia* (Smalley *et al*. 2016; Burke *et al*. 2020). Thus, it is possible that patients with immature or compromised immune systems might be at a greater risk of developing clinical symptoms upon infections with *R. amblyommatis*. While tick saliva proteins are known to reduce local inflammation, pathogenic spotted fever rickettsiae actively modulate host immune responses to establish a replicative niche within the hostile intracellular environment of phagocytes recruited to the tick bite site (Chmelar *et al*. 2011; Marchal *et al*. 2011; Curto *et al*. 2019). Our *in vivo* data and clinical evidence indicate that infections with *R. amblyommatis* may invade endothelial cells and cause transient local vasculitis at the tick bite site until professional immune cells, such as macrophages or CD8-positive T cells, clear the invading pathogens. Future *R. amblyommatis* infection studies with *in vitro* tissue culture cells or *in vivo* animal models lacking specific immune components may reveal host cells and immune factors critical for controlling *R. amblyommatis* infections in humans.

We show here that three strains of *R. amblyommatis* share highly similar genome sequences and have conserved genes shown to be important for spotted fever pathogenesis in animal infection models. Cross-comparison of spotted fever *Rickettsia* genome sequences revealed multiple gene rearrangements and gene deletions in *R. conorii* and *R. rickettsii*, presumably enhancing their intracellular parasitism to cause spotted fever pathogenesis in mammalian hosts. While all three rickettsial species are predicted to produce comparable numbers of proteins (1229–1269), *R. amblyommatis* had extra copies of pseudogenes and genetic areas undergoing genome reduction. The precise molecular mechanisms whereby genes and genomes deteriorate in *Rickettsia* remain mostly unresolved (Blanc *et al*. 2007). A large proportion of *A. americanum* ticks is infected with *R. amblyommatis* in the USA, often exceeding 60% in questing ticks (Jiang *et al*. 2010; Parola *et al*. 2013; Karpathy *et al*. 2016; Sanchez-Vicente *et al*. 2019). *Rickettsia amblyommatis* is also closely associated with other species within the genus *Amblyomma* in South America (Nunes Ede *et al*. 2015; Mastropaolo *et al*. 2016). *Amblyomma* ticks are also capable of transmitting multiple human pathogens, including *Ehrlichia chaffeensis* (human monocytic ehrlichiosis), *Ehrlichia ewingii* (human granulocytic ehrlichiosis), *Coxiella burnetii* (Q fever), *Francisella tularensis* (tularemia), *R. parkeri* (spotted fever rickettsiosis) and *R. rickettsii* (RMSF) (Childs and Paddock 2003). In addition, *Amblyomma* ticks have been implicated in a life-threatening allergic reaction to α-Gal, also known as meat allergy (Araujo *et al*. 2016; Hashizume *et al*. 2018; Mitchell *et al*. 2020). Recent tick surveys revealed that numerous species in the genus *Amblyomma* ticks have expanded and invaded into the new geographical regions, creating a potential public health emergency (Childs and Paddock 2003). At the same time, this provides us with a unique opportunity to isolate and characterize multiple *R. amblyommatis* strains parasitizing *Amblyomma* ticks in historical areas (regions with warm and humid climates, e.g. South and Central USA) and compare them with those surviving within *Amblyomma* ticks invading new geographical areas (regions with cold and dry climates, e.g. Northeastern USA). Cross-comparison of genome sequences of *R. amblyommatis* will provide insights into the molecular and genetic processes of reductive genome evolution in *Rickettsia*, their interactions with other bacterial pathogens of medical importance, their adaptation to new tick vectors and environment, as well as their impact on vector competence and disease transmission.
